# Supplementary material for: Cystathionine Gamma Lyase Is Regulated by Flow and Controls Smooth Muscle Migration in Human Saphenous Vein
Source: Antioxidants (Basel). 2023 Sep 7;12(9):1731. doi: 10.3390/antiox12091731 (PMC10525225; doi:10.3390/antiox12091731)
Supplement: Supplementary file 1 [file antioxidants-12-01731-s001.zip › antioxidants-2529096-supplementary.pdf]

Supplementary Material

Supplementary Table S1: Antibodies

Target antigen	Vendor	Catalog #	Working concentration	RRID
**Goat anti-Rabbit Alexa Fluor 594**	Thermo Fisher Scientific, Ecublens, Switzerland	A32740	1/250 (ICC)	AB_2762824
**Goat anti-Rabbit Alexa Fluor 488**	Thermo Fisher Scientific, Ecublens, Switzerland	A11034	1/500 (ICC)	AB_2576217
**Anti-Rabbit HRPO**	Thermo Fisher Scientific, Ecublens, Switzerland	31460	1/20000 (WB)	AB_228341
**Anti-mouse HRPO**	Jackson ImmunoResearch Labs, Thermo Fisher Scientific, Ecublens, Switzerland	115-035-146	1/15000 (WB)	AB_2307392
**BrdU**	BD Biosciences, Allschwil, Switzerland	555627	1/200 (ICC)	AB_10015222
**VWF**	DAKO, Agilent Technologies (Schweiz) AG, Basel, Switzerland	A0082	1/20000 (IHC-P)	
**eNOS**	BD Biosciences, Allschwil, Switzerland	610297	1/1000 (WB)	
**CSE**	Proteintech, LubioScience GmbH, Zurich, Switzerland	12217-1-AP	1/1000 (WB)<br>1/250 (IHC-P)	AB_2087497
**CBS**	Santa Cruz Biotechnology, Heidelberg, Germany	sc-133154	1/1000 (WB)<br>1/100 (IHC-P)	AB_2244094
**3-MST**	Sigma-Aldrich, Merck KGaA, Darmstadt, Germany	HPA001240	1/500 (IHC-P)	AB_1079408
**3-MST**	Novus Biologicals, Bio-Techne AG,Zug, Switzerland	NBP1-82617	1/2000 (WB)	AB_11014969

Supplementary Table S2: Reagents

Kit/product	Vendor	Catalog #
BrdU	Sigma-Aldrich, Merck KGaA, Darmstadt, Germany	B5002
Immobilon Western Chemiluminescent HRP Substate	Millipore, Merck KGaA, Darmstadt, Germany	WBKLS0050
Immobilon-P transfer membrane	Millipore, Merck KGaA, Darmstadt, Germany	IPVH00010
EnVision® + Dual Link System-HRP (DAB+)	DAKO (now Agilent Technologies (Schweiz) AG, Basel, Switzerland)	K4065
Vectashield Antifade Mounting Medium with DAPI	Vector Laboratories, Reactolab, Waldeck, Switzerland	H-1200
Pierce reversible protein Stain Kit for PDGF membranes	Thermo Fisher Scientific, Ecublens, Switzerland	24585
RPMI-1640 Glutamax I	Gibco, Thermo Fisher Scientific, Ecublens, Switzerland	61870-010
Gelatin type B	Sigma-Aldrich, Merck KGaA, Darmstadt, Germany	G9391
TriPure™	Roche Diagnostics, Rotkreuz, Switzerland	11667157001
DAz-2	Cayman Chemicals, Chemie Brunschwig AG Basel, Switzerland	13382
Cyanine 5.5 alkyne	Lumiprobe, Waldeck, Switzerland	C70B0
4-Chloro-7-Nitrobenzofurazan	Sigma-Aldric, Merck KGaA, Darmstadt, Germany h	163260
SF_7-AM fluorescent probe	Sigma-Aldrich, Merck KGaA, Darmstadt, Germany	748110
Resorcine-Fuchsine Weigert	Reactolab, Waldeck, Switzerland	2E-030
Hematoxylin crist.	Merck KGaA, Darmstadt, Germany	1.04302.0025
Acid Fuchsin	Sigma-Aldrich, Merck KGaA, Darmstadt, Germany	F8129
Picric acid	Sigma-Aldrich, Merck KGaA, Darmstadt, Germany	197378
Neo-Clear	Merck KGaA, Darmstadt, Germany	1.09843.5000
Ethanol absolu	Merck KGaA, Darmstadt, Germany	1.00983.2500
Phosphate Buffered Saline (PBS)	Laboratorium Dr. G. Bichsel AG, Unterseen, Switzerland	100 0 324
L-cysteine	Sigma-Aldrich, Merck KGaA, Darmstadt, Germany	C7352
Sodium dodecyl sulfate (SDS)	Promega AG, Dübendorf, Germany	H5114
Tween-20	Applichem GMBH, Darmstadt, Germany	A1389
Triton x-100	Sigma-Aldrich, Merck KGaA, Darmstadt, Germany	T8787
Lead Acetate trihydrate	Acros Organics (Now Thermo Fisher Scientific)	423841000
Pyridoxal 5'-phosphate monohydrate	Sigma-Aldrich, Merck KGaA, Darmstadt, Germany	82870
Bovine Serum albumin (BSA)	Applichem GMBH, Darmstadt, Germany	A1391
DC™ Protein Assay Kit I	Bio-Rad Laboratories, Cressier, Switzerland	5000111
Fast SYBR™ Green Master Mix	Applied Biosystems; Agilent Technologies (Schweiz) AG, Basel, Switzerland	4385618
PrimeScript RT Reagent Kit (Perfect Real Time)	Takara Bio Europe SAS, Saint-Germain-en-Laye, France	RR037B

Supplemental Table S3: qPCR oligo primers

Primer Name	Sequence
**Human CTH**	Fw: CCAGCACTCGGGTTTTGAAT<br>Rv: TACTTAGCCCCATCCAGTGC
**Human CBS**	Fw: ATGGTGACGCTTGGGAACAT<br>Rv: GGCGGATCTGTTTGAACTGC
**Human MPST**	Fw: CCGAGACGGCATTGAACCT<br>Rv: CCTGGCTCAGGAAGTCTGTG
**Human NOS3**	Fw:GCCGGAACAGCACAAGAGTTA<br>Rv:CCCTGCACTGTCTGTGTTACT
**Human HMOX1**	Fw: AGGAGGTCATCCCCTACACA<br>Rv: GCGGTAGAGCTGCTTGAACT
**Human TXN1**	Fw: AGCCTTTCTTTCATTCCCTCTC<br>Rv: ACATCCTGACAGTCATCCACAT
**Human ATF4**	Fw: TTAAGCCATGGCGCTTCTCA<br>Rv: ATTTCGGTCATGTTGCGGTG
**Human KLF2**	Fw: TATCTTGCCGTCCTTTGCCA<br>Rv: CGTTGTTTAGGTCCTCATCCG

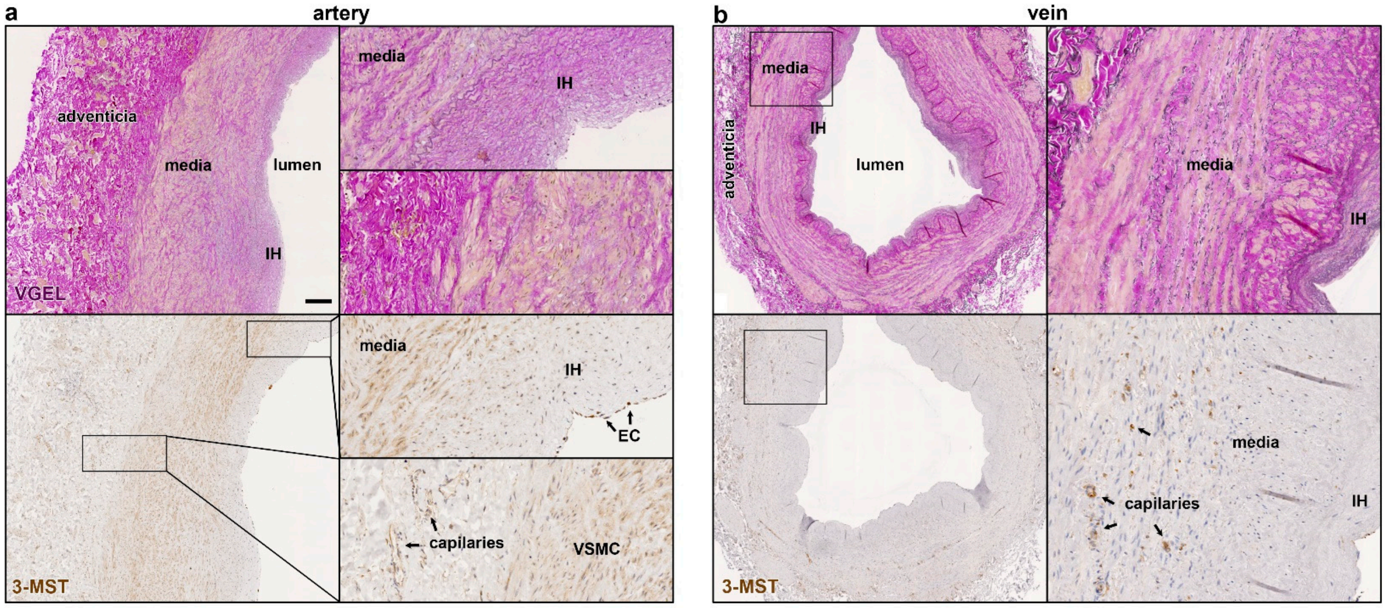

Figure S1. 3MST is mainly expressed in endothelial cells in large artery and vein segments.

Representative VGEL staining or 3-MST immunostaining in freshly isolated human aorta (**a**) and saphenous vein (**b**). Images are representative of 4 to 5 different arteries/veins. IH: intimal hyperplasia. Insets are 3-fold magnifications.

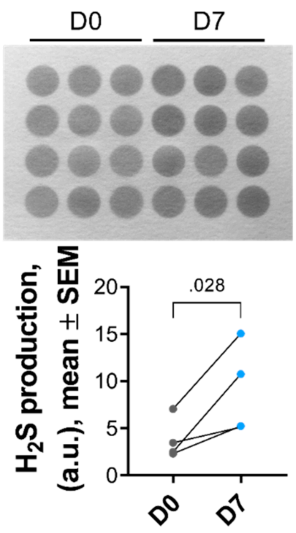

Figure S2. Static vein culture promotes CSE-derived H_2S production. Lead acetate assay of freshly isolated human vein segments (D0) or after 7 days (D7) in static culture. 4 different veins/patients in triplicates. P value as determined by bilateral paired t-test.

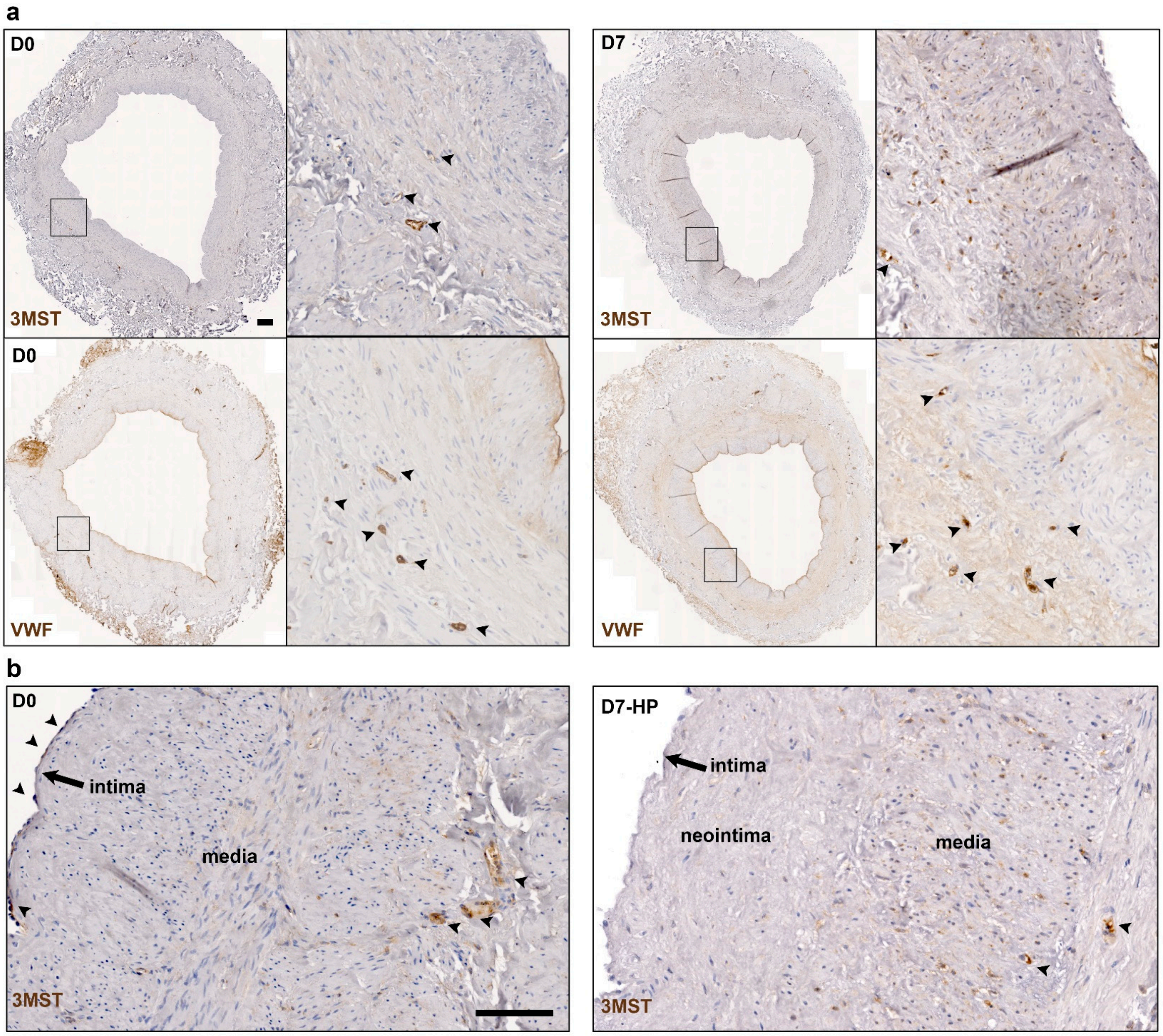

Figure S3. Static vein culture promotes 3-MST expression in the media of saphenous veins

(**a**) 3-MST immunostaining in freshly isolated human saphenous vein (D0) or after 7 days (D7) in static culture. Representative of 5 different veins/patients. Insets are 5-fold magnifications form black boxes. Scale bar =100 µm. (**b**) 3-MST immunostaining in freshly isolated human saphenous vein (D0) or after 7 days in culture under arterial conditions (D7-HP). Scale bar =200 µm.

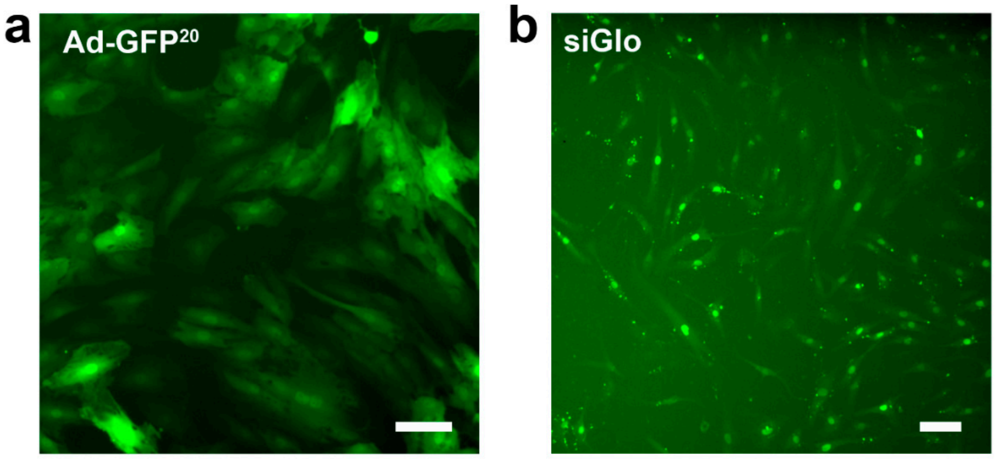

Figure S4. Infection and transfection efficiency. (**a**) Live-cell imaging of GFP fluorescence 48h post VSMC infection with an adenovirus encoding GFP (AdGFP) at MOI 20. (**b**) Live-cell imaging of siGlo fluorescence 24h post VSMC. Images are representative of 4 independent experiments. Scale bar100 µm.

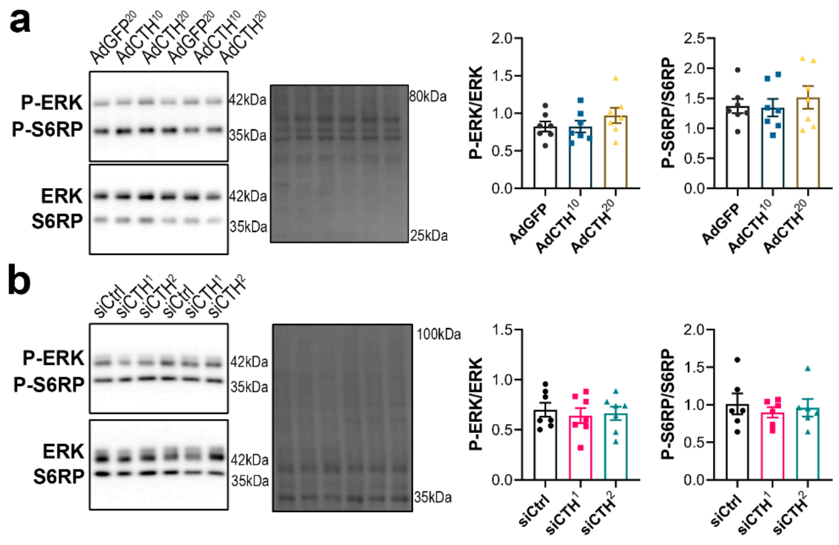

Figure S5. CSE modulation does not regulate P-ERK or P-S6RP levels. (**a**) VSMC were infected with an adenovirus encoding GFP (AdGFP) or CSE (AdCTH) at MOI 10 or 20, as indicated. (**b**) VSMC were transfected with a control siRNA (siCtrl) or two distinct CTH siRNA (siCTH 1 and 2), as indicated. (**a**,**b**) Representative Western blot analyses and quantitative assessment of P-ERK over total ERK and P-S6RP over S6RP. Data shown as mean ± SEM of 6 to 7 independent experiments. No statistical differences as determined by repeated measures one-way ANOVA.
